# The Correlation Between Pre-treatment Fluorodeoxyglucose Positron Emission Tomography/Computed Tomography Parameters and Clinical Prognostic Factors in Pediatric Hodgkin Lymphoma

**DOI:** 10.4274/mirt.94914

**Published:** 2017-02-01

**Authors:** Ebru Tatcı, İnci Uslu Biner, Suna Emir, Hikmet Gülşah Tanyıldız, Özlem Özmen, Engin Alagöz, Atila Gökçek, Gürses Şahin

**Affiliations:** 1 Atatürk Chest Diseases and Thoracic Surgery Training and Research Hospital, Department of Nuclear Medicine, Ankara, Turkey; 2 Ankara Children’s Hematology Oncology Training and Research Hospital, Department of Pediatric Hematology Oncology, Ankara, Turkey; 3 Dr. Sami Ulus Maternity and Children’s Health and Diseases Training and Research Hospital, Department of Pediatric Oncology and Hematology, Ankara, Turkey; 4 Gülhane Training and Research Hospital, Department of Nuclear Medicine, Ankara, Turkey; 5 Atatürk Chest Diseases and Thoracic Surgery Training and Research Hospital, Department of Radiology, Ankara, Turkey

**Keywords:** Fluorodeoxyglucose positron emission tomography/computed tomography, Hodgkin lymphoma, Standardized uptake value, metabolic tumor volume

## Abstract

**Objective:**

To compare standardized uptake values (SUV) derived from pre-treatment 18F-fluorodeoxyglucose (FDG) positron emission tomography/computed tomography (PET/CT) imaging and clinical prognostic factors in pediatric patients with Hodgkin lymphoma (HL).

**Methods:**

Pre-treatment FDG PET/CT findings of 28 children with HL were evaluated in this retrospective study. Metabolic tumor volume (MTV), SUV_max_ normalized by weight (SUVw_eight_), lean body mass (SUV_lbm_), body surface area (SUV_bsa_) and plasma glucose levels of tumors (SUV_glucose_) were calculated using pre-treatment FDG PET/CT scan images. These metabolic parameters were correlated with clinical factors [age, sex, number of lymph node groups, presence of splenic involvement, bulky mediastinal disease, Ann Arbor stage, serum white blood cell (WBC) count, erythrocyte sedimentation rate (ESR), serum albumin and hemoglobin levels].

**Results:**

SUV_bsa_, SUV_lbm_, SUV_weight_, SUV_glucose_ and MTV were higher in patients with stage III-IV disease, bulky tumor and ≥3 lymph node groups (p<0.05). SUV_bsa_ and SUV_glucose_ were higher in patients with splenic involvement (p<0.05). There was no significant correlation between these metabolic parameters and sex, ESR, levels of albumin and WBC (p>0.05). SUV_bsa_ and SUV_lbm_ were higher in patients with anemia (p<0.05). Additionally, significant increases were detected in SUV_weight_, MTV, and SUV_glucose_ with increasing age (p=0.005, p=0.027, and p=0.009, respectively). SUV_bsa_ and SUV_lbm_ had no significant correlation with age (p>0.05).

**Conclusion:**

Metabolic parameters derived from pre-treatment FDG PET/CT may have an important role in predicting high-risk disease in patients with HL. Also, SUV_bsa_ and SUV_lbm_ may be better markers than SUV_weight_ in the quantitative evaluation of FDG PET/CT scans in pediatric patients.

## Abstract (tr)

**Amaç:**

Hodgkin lenfoma (HL) tanısı konmuş çocuk hastalarda klinik prognostik faktörler ile tedavi öncesi ^18^F-florodeoksiglukoz (FDG) pozitron emisyon tomografi/bilgisayarlı tomografi (PET/BT) görüntülemeden elde edilen standart tutulum değerlerini (SUV) karşılaştırmaktır.

**Yöntem:**

Bu retrospektif çalışmada HL tanılı 28 çocuk hastanın FDG PET/BT bulguları değerlendirildi. Metabolik tümör volümü (MTV), kiloya (SUV_kilo_), yağsız vücut kitlesine (SUV_lbm_), vücut yüzey alanına (SUV_bsa_) and plazma glukoz seviyesine (SUV_glukoz_) göre normalize edilmiş SUV_maks_ değerleri tedavi öncesi FDG PET/CT görüntüleri kullanılarak hesaplandı. Bu metabolik parametrelerin klinik faktörler [yaş, cinsiyet, lenf nodu grup sayısı, dalak tutulumu, büyük mediastinal hastalık, Ann Arbor evreleme, serum lökosit sayımı (WBC), eritrosit sedimentasyon hızı (ESH), serum albumin ve hemoglobin seviyesi] ile ilişkisi araştırıldı.

**Bulgular:**

SUV_bsa_, SUV_lbm_, SUV_kilo_, SUV_glukoz_ ve MTV evre 3-4 hastalığı, büyük tümörü ve ≥3 lenf nodu grubu olan hastalarda daha yüksekti (p<0,05). SUV_bsa_ ve SUV_glukoz_ dalak tutulumu olan hastalarda daha fazlaydı (p<0,05). Bu metabolik parametreler ile cinsiyet, ESR, albumin ve WBC seviyeleri arasında önemli bir ilişki yoktu (p>0,05). SUV_bsa_ ve SUV_lbm_ anemisi olan hastalarda daha yüksekti (p<0,05). Ek olarak yaş arttıkça SUV_kilo_, MTV ve SUV_glukoz_’da önemli artış olduğu saptandı (sırasıyla; p=0,005, p=0,027 ve p=0,009). SUV_bsa_ ve SUV_lbm_ ile yaş arasında önemli korelasyon yoktu (p>0,05).

**Sonuç:**

HL tanılı hastalarda tedavi öncesi FDG PET/BT’den elde edilen metabolik parametreler yüksek riskli hastalığı tahmin etmede önemli bir rol oynayabilir. Ayrıca, pediatrik hastalarda FDG PET/BT’nin kantitatif değerlendirilmesinde SUV_bsa_ ve SUV_lbm_, SUV_kilo_’dan daha iyi belirteç olabilir.

## INTRODUCTION

Currently, more than 80% of patients with Hodgkin lymphoma (HL) can be cured by contemporary treatment methods. The present major problem with HL is the long-term complications of treatment. Children who have been treated for HL have a higher risk of developing secondary tumors, cardiac events, and infections (1). Prognostic factors are considered during treatment planning to decrease the side effects and the likelihood of recurrence or treatment resistance (2). The most unfavorable prognostic factors according to the International Prognostic Score are stage 4 disease, age ≥45 years, hemoglobin <10.5 g/dl, albumin <4.0 g/dl, white cell count (WBC) ≥15,000/µl, and lymphocyte level <600/µl or <8%, respectively. Also, presence of B symptoms, high erythrocyte sedimentation rate (ESR), male sex, higher number of involved nodal sites, and bulky-mass tumors are additional factors associated with an increased risk of relapse (3).

It has been reported in many studies that FDG PET-CT is a very useful imaging modality in the primary staging, restaging, assessment of treatment response and evaluation of residual masses of lymphomas (4,5,6,7). Standardized uptake value (SUV) is used traditionally for the definition of metabolic activity in FDG PET imaging. The patient’s body weight is usually employed as the body size measurement during the calculation of SUV. However, lean body mass or body surface area may be preferred for body size measurement by some authors. SUV is also affected by blood glucose level, post-injection uptake time, image resolution, image reconstruction parameters, and volume-of-interest-definition (8,9). Additionally, the maximum SUV (SUV_max_) is only measured by the highest image pixel in the tumor regions and doesn’t show the metabolic activity of the entire tumor. Metabolic tumor volume (MTV) is another FDG PET/CT parameter, which is the measurement of the tumor volume with increased metabolism. It has been reported that MTV could play an important role in predicting survival in various malignancies (10,11). The purpose of this study was to evaluate the role of FDG-PET/CT in staging pediatric HL, and to establish if the metabolic parameters of pre-treatment FDG PET/CT correlated with clinical prognostic factors in pediatric HL patients. If so, metabolic parameters of pre-treatment FDG PET/CT might have a role in predicting treatment failure. Additionally, we evaluated the correlation of age and metabolic parameters in pediatric patients.

## MATERIALS AND METHODS

### Patients

Twenty-eight HL patients who underwent pre-treatment FDG PET/CT examinations between May 2009 and December 2014 were included in this retrospective study. Informed consent was waived due to the retrospective nature of the study. Patients older than 18 years were excluded. The study was approved by the Institutional Ethics Committee. The histologic classifications were established according to the standard WHO classification scheme (12). Results of bone marrow biopsy (BMB), levels of WBC, albumin and hemoglobin values and ESR were recorded. After completion of therapy, patients were followed by physical examination, laboratory analyses, chest radiographs, ultrasonography, CT or FDG PET/CT scans.

### Fluorodeoxyglucose Positron Emission Tomography/Computed Tomography Imaging

PET/CT imaging was performed forty-five to sixty minutes after intravenous injection of 87.69-414.77 MBq (2.37-11.21 mCi) of FDG with a Siemens Biograph 6 HI-REZ integrated PET/CT scanner (Siemens Medical Solutions, Knoxville, TN, USA). All patients fasted for at least six hours before PET/CT imaging without water restriction. The blood glucose levels of patients were confirmed to be less than 180 mg/dL before FDG injection. Low-dose whole-body CT was used for attenuation correction. PET/CT data were acquired from the top of the skull to the upper thigh.

### Fluorodeoxyglucose Positron Emission Tomography/Computed Tomography Analysis

### Visual Analyses

Pre-treatment PET/CT images were retrospectively evaluated by two experienced nuclear medicine physicians and one radiologist. The number of lymph node groups was determined. A splenic FDG uptake greater than hepatic uptake was considered as splenic involvement. The marrow was considered as abnormal when the uptake was equal to or greater than that of the liver. Bulky disease was defined as presence of a lymph node mass greater than 0.33 of the maximum intrathoracic cavity width. The intensity of FDG activity within the bone marrow (BM) was evaluated visually. BMB results were used as the gold-standard for staging. The stage of lymphoma was assessed according to the Ann-Arbor classification (13).

### Semi-quantitative Analyses

SUV_max_ and MTV values were obtained from pre-treatment FDG PET/CT images for semi-quantitative evaluation. The SUV_max_ corrected for body weight (SUV_weight_) was measured within the hottest tumor lesion according to the formula (14):

SUV_weight_ = Tissue concentration (MBq/ml) / Injected dose (MBq)/ weight (g)

The SUV_max_ normalized for lean body mass (SUV_lbm_), and body surface area (SUVbsa) were calculated using the following equations (14):

SUV_Ibm_ = Tissue concentration (MBq/ml) / Injected dose (MBq)/ lbm (kg)

LBM (male) (kg)=(1.1×weight) (kg)-120 [weight (kg)/height (cm)]^2^

LBM (female) (kg)=(1.07×weight) (kg)-148 [weight (kg)/height (cm)]^2^

SUV_bsa_ = Tissue concentration (MBq/ml) / Injected dose (MBq)/ BSA (m^2^)

BSA (m^2^)=0.007184×weight (kg)^0.425^×height (cm)^0.725^

SUV values were also corrected for blood glucose level using an established formula, assuming a normal blood glucose level of 5.55 mmol/L (100 mg/dL) (14):

SUV_glucose_ = SUV_max_×blood glucose (mmol/L) / 5.55 mmol/L

MTV of each hypermetabolic tumor focus was automatically calculated by the software program and MTV of each tumor was summated. The threshold intensity value used in this study was 40% of maximal SUV of each tumor as validated in several previous studies (15,16).

### Statistical Analyses

The SPSS 20 software was used for statistical analysis. Comparisons of SUV levels (SUV_weight_, SUV_bs_a, SUV_lbm_, SUV_glucose_, MTV) and clinical parameters (sex, number of lymph node groups, presence of splenic involvement, bulky disease, Ann Arbor stage, serum levels of albumin, WBC, ESR and hemoglobin) were performed using the independent test or Mann-Whitney U test. All quantitative values are given as mean±standard deviation (SD). Pearson’s correlation coefficients were used to evaluate the correlation between PET parameters and age. A statistically significant difference was defined as a p value <0.05.

## RESULTS

### Patients

The characteristics of the patients are presented in Table 1. A total of 28 patients with a mean age ±SD of 9.39±4.2 y (male/female: 17/11) were enrolled in this study. Eleven (39.3%) patients had nodular sclerosis subtype and 15 (53.6%) had mixed cellularity subtype. Histologic subtype remained unclassified in two patients. Of all 28 patients, 18 (64.2%) had undergone BMB.

The laboratory findings of the patients are summarized in Table 2. Among all 28 patients, hemoglobin was <10.5 g/dl in nine patients (32.1%), WBC was ≥15000/ml in 11 patients (39.3%), albumin was <4 g/dl in 16 patients (57.1%), and ESR was ≥50 mm/hr in 13 patients (46.4%). All of the patients underwent three to six courses (6-12 cycles) of chemotherapy with or without radiation therapy after the initial PET/CT scanning. The mean±SD clinical follow-up period was 29±14 months. All 28 patients were in remission at the last follow up, but one child died of infection. Recurrence was not detected in the follow-up.

### Fluorodeoxyglucose Positron Emission Tomography/Computed Tomography Analyses

### Visual Analyses

Pre-treatment FDG PET/CT findings are summarized in Table 2. Bulky mediastinal disease was detected in 15 of the 28 (53.5%) patients. While there were <3 lymph node groups in 28.6% of the 28 patients (n=8), ≥3 lymph node groups were found in 71.4% (n=20). Splenic involvement was detected in 32.1% patients (n=9). Eight patients had stage 1-2 (28.6%) disease, and 20 patients had stage 3-4 (71.4%) disease.

**Figure 1:**
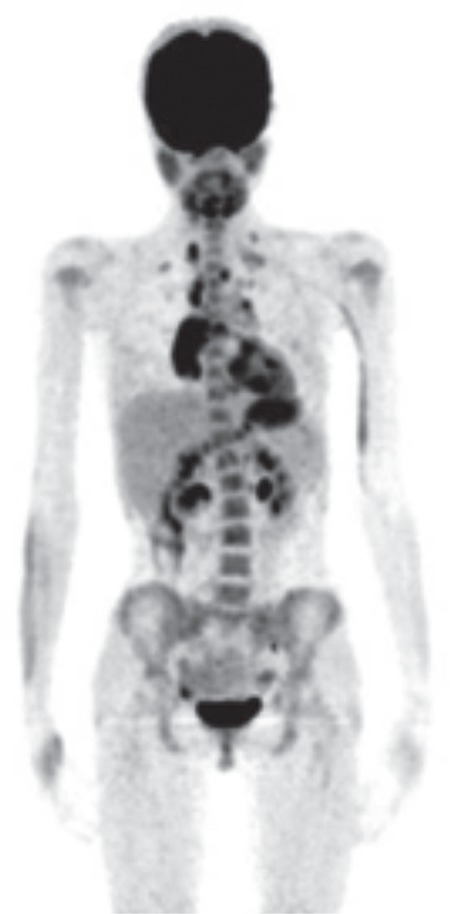
The maximum intensity projection image. Increased diffuse bone marrow fluorodeoxyglucose uptake in the axial system, and fluorodeoxyglucose uptake by supra-diaphragmatic lymph nodes are displayed. Bone marrow biopsy was negative

**Figure 2:**
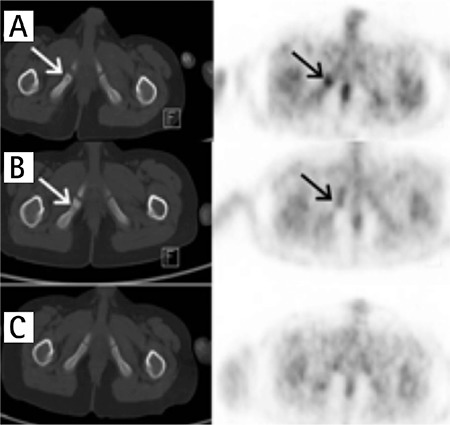
A) Axial positron emission tomography/computed tomography images of the pelvis in the bone window setting showed radiolucency and enlargement at the right ischium and increased focal fluorodeoxyglucose uptake at that area. B) Sclerosis at the same localization and decreased fluorodeoxyglucose uptake four months after therapy were seen. C) After 12 months of therapy no abnormal findings were detected on positron emission tomography/computed tomography scan (arrows)

Increased diffuse BM FDG uptake in the axial skeleton was seen in 12/28 (42.8%) patients. BMB was performed in 10 of these 12 (83.3%) patients. Among ten patients with diffuse BM uptake, nine patients had negative BMB results (Figure 1). Only one patient showed positive lymphoma involvement in BMB. There was no increase in BM FDG uptake in 16 of 28 (57.1%) patients. BMB was performed in seven of 16 (43.7%) whose BMB result was negative. Additionally radioluceny and enlargement at the localizations of ischiopubic synchondrosis (IPS) were detected in 4 of the 28 (14.2%) patients. Intense FDG uptakes were seen in these areas (SUV_max_ range 1.17-3.10). These patients didn’t suffer from any symptoms such as groin pain and restriction in the movement of the hip joint. Since these focal FDG uptakes weren’t identified in follow-up PET/CT scans, they were evaluated as the asymmetric ossification pattern of the IPS rather than malignant bone infiltration (Figure 2).

### Quantitative Analyses

SUV_bsa_, SUV_lbm_, SUV_weight_, SUV_glucose_, and MTV values were higher in patients with stage 3-4 disease, a bulky tumor, and ≥3 lymph node groups (p<0.05) (Table 2). SUV_bsa_ and SUV_glucose_ were higher in patients with splenic involvement (p<0.05). There was no significant correlation between metabolic parameters and a) sex, b) ESR, c) albumin level, and d) WBC level (p>0.05). Hemoglobin level lower than 10.5 g/dL was associated with higher SUV_bsa_ and SUV_lbm_ (p<0.05). While SUV_bsa_ and SUV_lbm_ (p>0.05) did not relate to age; SUV_weight_, MTV, and SUV_glucose_ values were found to be significantly correlated with age (p=0.005, p=0.027, p=0.009 and r=0.519, r=0.506, r=0.504, respectively).

## DISCUSSION

It was previously demonstrated in some studies in the literature that the amount of FDG accumulation is an important prognostic factor in various malignant tumors (17,18). Ceriani et al. (19) reported that elevated MTV was significantly associated with worse progression-free and overall survival in patients with primary mediastinal (thymic) large B-cell lymphoma. Suh et al. (20) showed that pre-treatment FDG PET could predict treatment response and survival outcomes in patients with extranodal natural killer/T-cell lymphomas of the head and neck. Similarly, we identified a significant association between quantitative FDG uptake and clinical prognostic factors in pediatric patients with HL. The quantitative parameters were higher in patients with stage 3-4 disease, a bulky tumor, and ≥3 lymph node groups (p<0.05), which are the clinical parameters that reflect tumor burden. Intensity of tumor cells is one of the most important parameters to determine the efficacy of treatment in HL (21). High SUV_max_ and MTV can indicate poorer survival in patients with HL. Intensive therapy can be administered in these patients. However, in the presence of low FDG uptake, overtreatment can be avoided. The present study has several limitations. Cure was achieved in 27 of the 28 patients at the end of therapy and recurrence was not detected on follow-up. One child died of infection. So, progression-free survival and overall survival couldn’t be determined in this study.

Anemia, low albumin level, leukocytosis and high ESR level are particularly observed in advanced stage disease (3). HD is characterized by the presence of a low frequency of malignant cells known as Reed-Sternberg cells. The majority of the malignant tissues in HD constitutes a reactive cell infiltrate composed of variable proportions of lymphocytes, histiocytes, eosinophils, and plasma cells. Malignant Reed-Sternberg cells and reactive cells produce different cytokines. Close associations between elevation of cytokine levels in the plasma and presence of B symptoms, anemia, leukocytosis, high ESR and low serum albumin levels has been reported (22,23). Our study results indicate that hemoglobin level lower than 10.5 g/dL was associated with higher SUV_bsa_ and SUV_lbm_ (p<0.05). Higher SUV_bsa_ and SUBlbm may be related to higher tumor burden and higher plasma cytokine levels. However, we did not observe significant correlation between metabolic parameters and ESR, levels of albumin and WBC (p>0.05). Long-term follow-up studies with a larger group of patients may yield more satisfactory results.

The amountof SUV_weight_ changes was related to patient’s body weight. However, some reports show that SUV_lbm_ and SUV_bsa_ are less dependent on body weight than SUV_weight_ (8,9). Concordant with these reports, our study showed that while SUV_weight_, MTV and SUV_glucose_ increase with age (p<0.05), SUV_bsa_ and SUV_lbm_ did not significantly correlate with age (p>0.05). Changes with age can reduce the importance of SUV_weight_, MTV and SUV_glucose_ values as markers to discriminate malignant tumors from benign ones in children. Also, these parameters can be misguiding in evaluating therapy response. SUV_lbm_ and SUV_bsa_ may be preferred in the primary diagnosis, staging and follow up of malignancies in the pediatric population (24).

A diffuse homogeneous BM FDG uptake generally reflects hyperplastic BM caused by severe anemia, use of granulocyte colony stimulating factors, or chemotherapy. However, infiltration of tumor cells can also cause increased diffuse BM FDG uptake. Authors suggest that BMB is the gold standard in the staging of HL (25). However, focal FDG uptake can be adequate in the diagnosis of bone or BM involvement in HL (13). Nevertheless, physiologic FDG uptake patterns mimicking bone metastasis such as IPS should be taken into consideration while evaluating PET/CT scans in the pediatric population (26).

## CONCLUSION

Metabolic parameters derived from pre-treatment FDG PET/CT scanning can be valuable in predicting high-risk disease in pediatric HL. Also, SUV_bsa_ and SUV_lbm_ might be better markers than SUV_weight_ in the quantitative evaluation of FDG PET/CT scans in pediatric patients. However, prospective studies with a larger group of patients are needed to obtain more reliable results.

## Figures and Tables

**Table 1:**
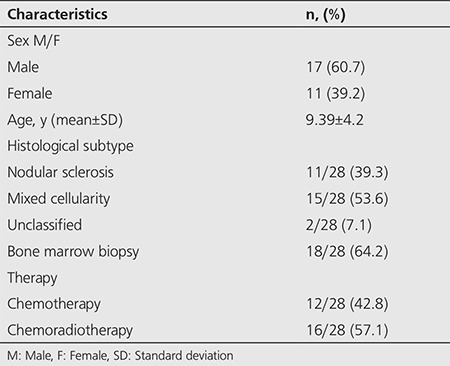
Patient characteristics

**Table 2:**
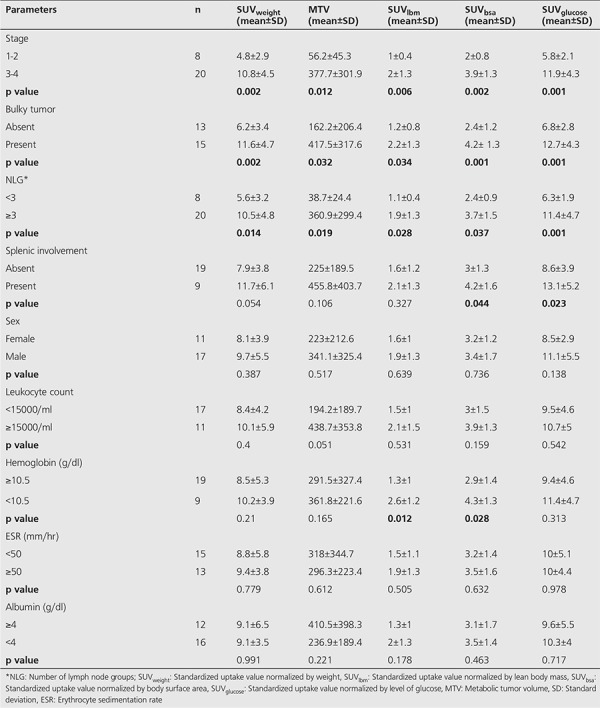
Fluorodeoxyglucose positron emission tomography/computed tomography parameters related to clinical prognostic factors
